# Combined Administration of Preoperative Intravitreal and Intraoperative Subretinal Recombinant Tissue Plasminogen Activator in Acute Hemorrhagic Age-related Macular Degeneration

**DOI:** 10.7759/cureus.7229

**Published:** 2020-03-10

**Authors:** Khaled Helaiwa, Lina R Paez, Peter Szurman, Kai Januschowski

**Affiliations:** 1 Ophthalmology, Knappschaft Hospital Sulzbach, Sulzbach, DEU; 2 Ophthalmology, Sulzbach Eye Hospital, An der Klinik, DEU; 3 Ophthalmology, University Eye Hospital Tübingen, Schleichstr, DEU

**Keywords:** intravitreal rtpa, neovascular age related macular degeneration, recombinant tissue plasminogen activator, submacular haemorrhage, vitrectomy

## Abstract

Purpose

To evaluate the efficacy of combining pre-operative intravitreal administration of recombinant tissue plasminogen activator (rTPA) followed by 23G pars plana vitrectomy with the subretinal administration of rTPA in the management of acute submacular hemorrhage (SMH) secondary to neovascular age-related macular degeneration (AMD).

Methods

This is a single-center case series report that included 14 patients with SMH secondary to neovascular AMD. All of them received preoperative intravitreal injection of 0.05 ml (50 µg) rTPA, followed on the next day by 23G pars plana vitrectomy with subretinal 0.1 ml (10 µg) rTPA administration and air tamponade.

Results

There was a significant (p=0.01) overall improvement in the visual acuity post-treatment (from 1.4±0.5 log MAR to 0.9±0.4). The mean overall change in the visual acuity post-treatment was 0.5±0.3 log MAR (mean % change=31.7±15.1). There was a significant (p=0.03) overall reduction in the central macular thickness post-treatment (896±608.1 µm to 497.2±196.0 µm). The mean overall change in the central macular thickness post-treatment was 398.8±458.1 µm (mean % change=38.1±18.1).

Conclusion

Combined treatment of 24 hours of preoperative administration of intravitreal rTPA followed the next day by vitrectomy and the administration of subretinal rTPA with air tamponade appeared to be effective as a prompt intervention in managing acute SMH secondary to neovascular AMD. However, similar studies with larger sample size and a control comparative group are warranted to further confirm these findings.

## Introduction

Submacular hemorrhage (SMH) is an accumulation of blood between the neurosensory retina and the retinal pigment epithelium (RPE) within the macular region [1]. SMH is a common and severe complication associated with exudative age-related macular degeneration (AMD). SMH results in retinal degeneration leading to extensive vision loss. SMH-induced retinal damage is due to the limited availability of nutrients to the retina, shrinkage of the outer retinal layers caused due to clot formation, and iron and hemosiderin toxicity [2]. The degeneration of the retina and the retinal pigment epithelium (RPE) ultimately causes acute vision loss [3]. The effects of SMH occur as early as 24 hours and results in the formation of macular scars due to the proliferation of fibrous tissue [4].

AMD patients taking anticoagulants are more prone to SMH damage, and if left untreated, the prognosis becomes very poor [2,4]. Several therapeutic modalities have been developed with the common aim of reducing or minimizing the damage to the sensory cells of the retina removing the submacular blood [5].

The most commonly practiced method of treating SMH is the injection of recombinant tissue plasminogen activator (rTPA) either subretinally or intravitreally combined with gas/air tamponade [6]. These methods have been reported to improve visual acuity [7]. rTPA dissolves the SMH and the gas displaces the SMH by either steam roller action or by gravity to a region where the SMH can be reabsorbed and the damage caused by SMH can be reduced [8]. It is been also shown that an air tamponade is as effective as gas, indicating that the duration of blood displacement is not a key factor [9].

Taking into consideration that the negative effects of the SMH occur as early as 24 hours and clot formation plays an important role in the pathogenesis of retinal damage, the timing of a quick and effective intervention would play an essential role for a better and optimal outcome. However, most of the patients usually present themselves to the clinic with a significant delay after the onset of symptoms and thus have a poor visual outcome; besides, mechanical clot extraction is associated with secondary complications such as proliferative vitreoretinopathy and retinal detachment. Intravitreal injection of rTPA would be one option because rTPA was shown to penetrate the retina and could not only resolve the blood clot but prevent contraction and scar formation. We would definitely agree that this option is less aggressive, however, in addition to the desired effects of intravitreal rTPA, we wanted to add the mechanical displacement and the more direct injection of rTPA into the accumulated submacular blood and to experience and evaluate the effectiveness of such prompt intervention in the treatment of acute SMH associated with neovascular AMD.

This work has earlier been presented as an abstract: Abstractband DOG 2019. Ophthalmologe. 2019, 116: 25-218. https://link.springer.com/article/10.1007%2Fs00347-019-0940-0

## Materials and methods

This was a single-center, prospective case series. Patients (n=14) with a submacular hemorrhage that presented to our clinic between June 2016 and February 2017 with a preoperative optical coherence tomography (OCT) evaluation and signed informed consent were included in the series. The inclusion criteria were: patients with acute SMH in the macular area with central subfoveal involvement secondary to neovascular AMD, hemorrhage not older than five days, a previously established diagnosis of AMD. The exclusion criteria were: patients with hemorrhage older than five days, hemorrhage outside the macular area, no previous established diagnosis of AMD, submacular hemorrhage due to choroidal neovascularisation (CNV) other than AMD (i.e., myopia, trauma, and angioid streaks). All of the patients were referred to our clinic from the primary health care ophthalmologists where they were initially diagnosed with neovascular AMD and were managed previously with several anti-vascular endothelial growth factor (anti-VEGF) intravitreal injections of bevacizumab. All of the patients had a diagnosis of advanced/late AMD at the time of referral.

The average time between the onset of symptoms and the diagnosis of submacular hemorrhage was 2.5 days (Range 2-4 days). The average time between the diagnosis of submacular hemorrhage and the administration of intravitreal rTPA was 1.3 days (Range 1-2 days).

We do agree that pre-hospital delay is a major issue in the management of SMH in AMD patients. Most of the patients come to the hospital with a significant delay after the onset of symptoms, and they are generally scheduled for surgery the next day or even later.

Preoperative examination consisted of a routine OCT volume scan of the macula region with a 30x30° pattern size (Pattern a number of 19 B-scans with a distance of about 235 - 240 µm (512-pixel x 496 pixels) using a spectral-domain OCT (Heidelberg Engineering, Heidelberg, Germany), best-corrected visual acuity, fundus photography, fundus autofluorescence, dilated funduscopy, and intraocular pressure (IOP) measurement. The recently described grading system based on the Early Treatment Diabetic Retinopathy Study (ETDRS) chart of the OCT result sheet was used to define the location in relation to the fovea, the volume scan was used to determine height and location in relation to the retinal pigment epithelium (RPE). The middle of the ETDRS chart is usually automatically centered on the fovea but was manually corrected if needed. This prospective study was approved by the local ethics committee and in accordance with the principles of the declaration of Helsinki. Follow-up examinations were done at four-six weeks at our clinic in the outpatient ward, after which they all received further Anti-VEGF treatments according to the Pro Re Nata regimen by their corresponding primary health care ophthalmologists.

rTPA preparation

1) Preparation of the stock solution (1 mg/ml)

10 mg rTPA (10 mg alteplase, (Actilyse®), Boehringer Ingelheim, Germany) dissolved in 10 ml of BSS (this results in a 1 mg/ml stock solution) may be portioned and frozen at -80 ° C.

2) Intravitreal injection preparation from the stock solution (1 mg/ml)

Injection of 0.05 ml of the stock solution = 50 μg rTPA intravitreal

3) Subretinal injection preparation from the stock solution (1 mg/ml)

0.5 ml of the stock solution is added to a sterile syringe with 5 ml BSS (this results in a 100 μg/ml in the 5 ml syringe)

Injection of approx. 0.1 ml = 10μg rTPA subretinal

Surgery

Patients were administered intravitreal rTPA 0.05 ml (50 µg) rTPA the day before surgery. The next day, pars plana vitrectomy was performed under a standard ophthalmic operating microscope (Lumera 7 CS microscope, Carl Zeiss Meditec Inc., Germany) by experienced surgeons only. A standard 23-gauge suture-less vitrectomy system was used (Eva, DORC, Dutch Ophthalmic Research Centre, the Netherlands) with the endoillumination set to 80% for all procedures. A complete core and peripheral vitrectomy was performed. Afterward, a subretinal injection of 0.1 ml (10 µg) rTPA was administered using a 41-gauge cannula (DORC, Dutch Ophthalmic Research Centre, the Netherlands). At the end of the surgery, a fluid-air exchange was made and an air tamponade was used. Finally, the infusion cannula was removed and the sclerotomies were sutured if necessary (Vicryl 7-0, Johnson & Johnson Intl., New Brunswick, New Jersey, United States of America). All of the patients experienced postoperative mild inflammation, redness, and mild pain, which lasted for only two to three days. The average postoperative intraocular pressure was 14.5 mmHg (Range 10-23 mmHg). None of the patients had immediate postoperative complications of re-bleeding, choroidal effusion/swelling, vitreous hemorrhage, or hypotony. All of the patients were discharged on the third postoperative day and were followed up regularly on a weekly basis up to four to six weeks postoperatively.

Statistical analysis

Data were presented as mean ± SD. A paired sample t-test was done to compare the outcomes of pre and post-treatment. A p-value of less than 0.05 was considered significant.

## Results

**Figure 1:**
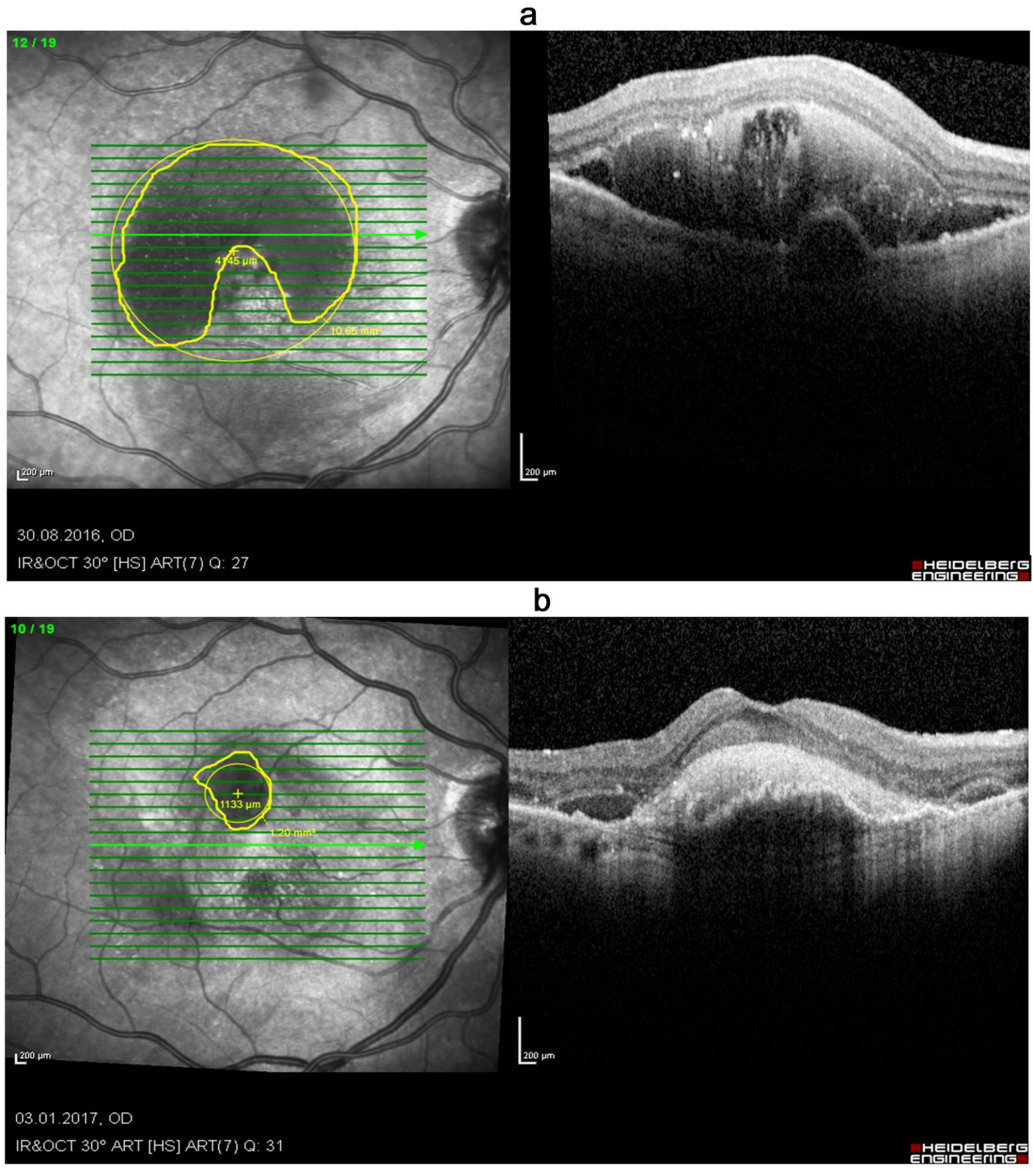
OCT images of an 83-year-old woman presented with acute SMH (a) before treatment (VA sc. 6/120) and (b) four weeks after treatment (VA sc. 6/60), showing a significant reduction of the bleeding area, but with poor visual outcome due to the formation of submacular scarring/fibrosis OCT: optical coherence tomography; SMH: submacular hemorrhage; VA: visual acuity

**Figure 2:**
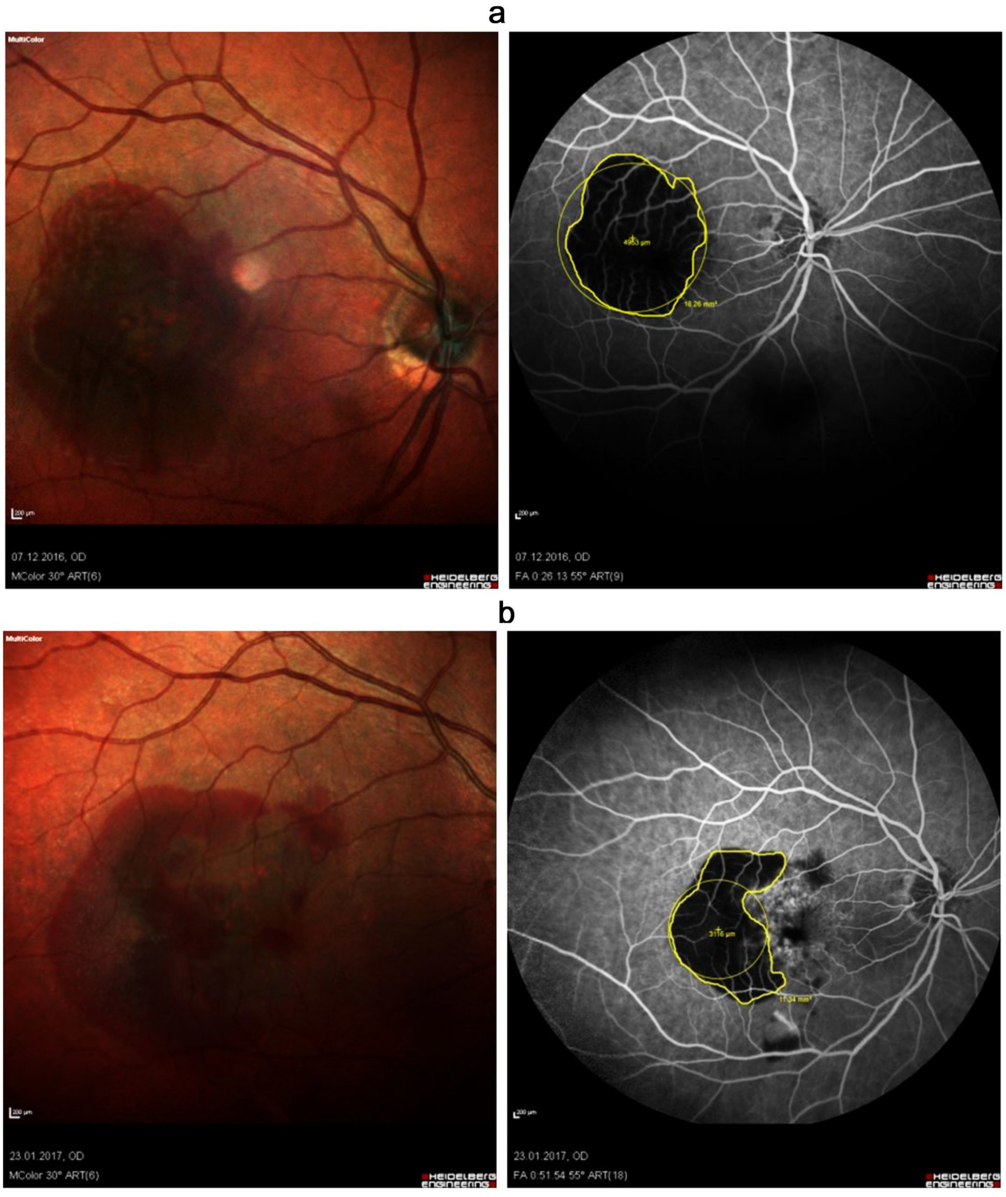
FFA of an 86-year-old woman with acute SMH (a) before treatment (VA sc. 6/600) and (b) six weeks after treatment (VA sc. 6/24), showing around a 35% reduction of the bleeding area but with good visual acuity improvement FFA: fundus fluorescein angiogram; SMH: submacular hemorrhage; VA: visual acuity

A total of 14 patients were included between June 2016 and February 2017 in the study. All patients provided written informed consent for the surgical procedure and the preoperative and postoperative examinations. There were four males and 10 females. The mean age of the patients was 83.9±5.2 years. All patients were pseudophakic. All patients had subretinal and sub-RPE (retinal pigment epithelium) hemorrhage. The average preoperative visual acuity was 1.4±0.5 logMAR (in decimal) preoperative. The preoperative mean central macular thickness was 896±608.1 µm and the mean Fundus Fluorescein Angiogram (FFA)/OCT hemorrhage area was 21.6±17.8 mm^2^ (Figures 1-2).

Using the grading system based on the ETDRS chart of the OCT, we found that five patients had a hemorrhage in the inferonasal, five patients had a hemorrhage in the supratemporal, and four patients had a hemorrhage in all four macular quadrants. All the patients had a subfoveal hemorrhage.

There was a significant (p=0.01) overall improvement in the visual acuity post-treatment (from 1.4±0.5 logMAR to 0.9±0.4). The mean overall change in the visual acuity post-treatment was 0.5±0.3 logMAR (mean % change=31.7±15.1) (Table 1).

**Table 1:** Change in visual acuity (VA) post treatment; visual acuity presented in LogMAR

Patient ID	VA pre-treatment	VA post-treatment	Change in VA post-treatment	% Change in VA post-treatment
1	0.7	0.4	0.3	42.9
2	1	0.6	0.4	40
3	1.7	1.3	0.4	23.5
4	2	1.6	0.4	20
5	1.7	1.3	0.4	23.5
6	2	0.6	1.4	70
7	1.4	1.3	0.1	7.1
8	1.6	1	0.6	37.5
9	1.3	1	0.3	23.1
10	0.6	0.5	0.1	16.7
11	1.6	1	0.6	37.5
12	1	0.7	0.3	30
13	0.8	0.5	0.3	37.5
14	2	1.3	0.7	35

There was a significant (p=0.03) overall reduction in the central macular thickness post-treatment (896±608.1 µm to 497.2±196.0 µm). The mean overall change in the central macular thickness post-treatment was 398.8±458.1 µm (mean % change=38.1±18.1) (Table 2).

**Table 2:** Change in central macular thickness post treatment; central macular thickness presented in µm

Patient ID	Central Macular Thickness Pre-treatment	Central Macular Thickness Post-treatment	Change in Central Macular Thickness Post-treatment	% Change in Central Macular Thickness Post-treatment
1	532	334	198	37.2
2	963	367	596	61.9
3	576	375	201	34.9
4	2878	1039	1839	63.9
5	1218	455	763	62.6
6	766	374	392	51.2
7	786	567	219	27.9
8	539	440	99	18.4
9	486	372	114	23.5
10	625	308	317	50.7
11	726	453	273	37.6
12	908	587	321	35.4
13	575	553	22	3.8
14	966	737	229	23.7

**Figure 3:**
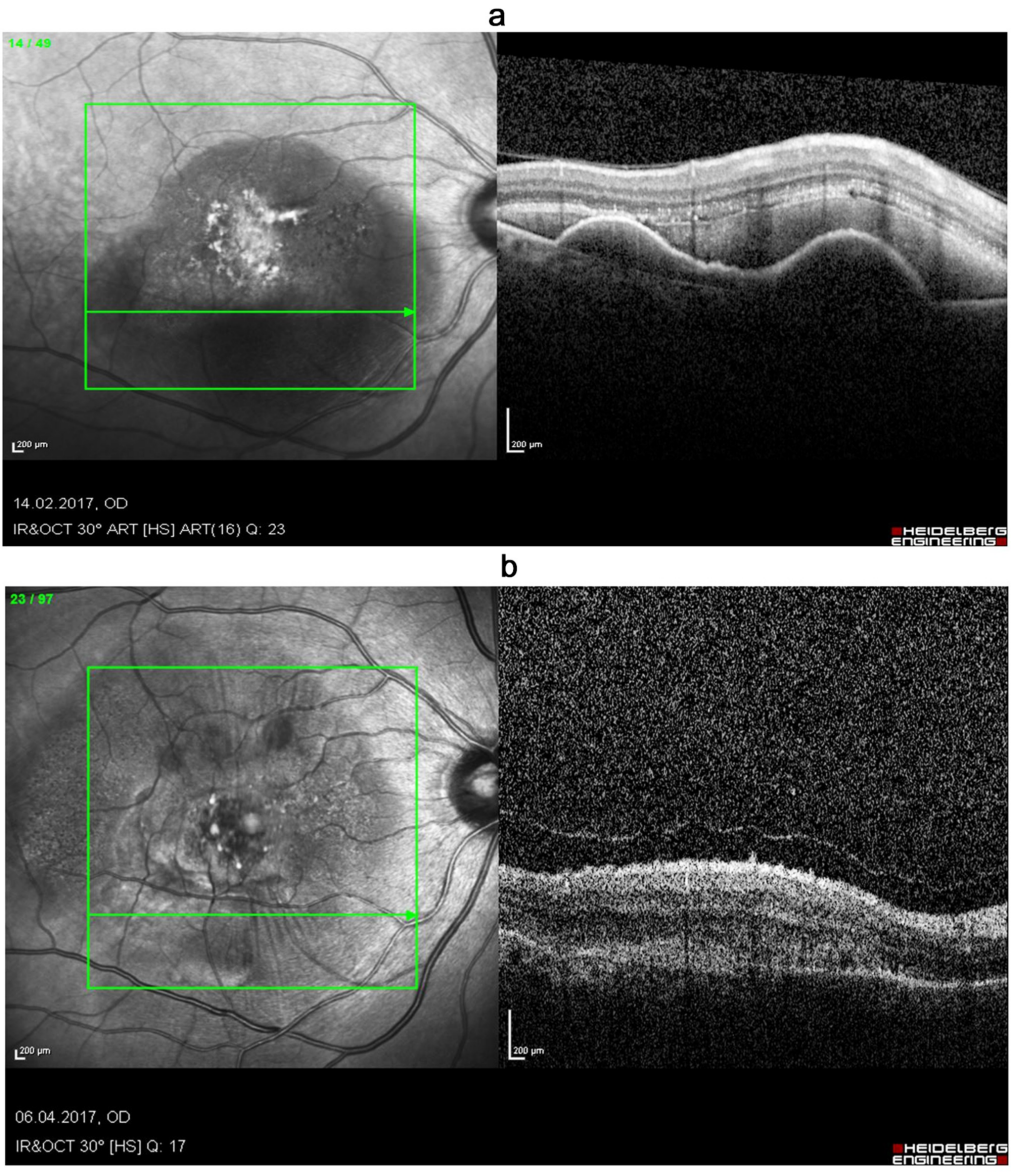
OCT images of an 85-year-old man presented with acute SMH (a) before treatment (VA sc. 6/120) and (b) six weeks after treatment (VA sc. 6/15), showing a significant reduction of the bleeding area, with good visual acuity improvement despite the formation of submacular scarring/fibrosis OCT: optical coherence tomography; SMH: submacular hemorrhage; VA: visual acuity

There was a significant (p=0.02) overall reduction in the submacular hemorrhage area post-treatment (21.6±17.8 mm^2^ to 7.5±10.7 mm^2^) (Figure 3).

The mean overall change in the submacular hemorrhage area post-treatment was 14.1±11.3 mm^2^ (mean % change=70.4±20.6) (Table 3).

**Table 3:** Change in hemorrhage area post-treatment; hemorrhage area presented in mm2

Patient ID	Hemorrhage Area Pre-treatment	Hemorrhage Area Post-treatment	Change in Hemorrhage Area Post-treatment	% Change in Hemorrhage Area Post-treatment
1	3.8	0.7	3.1	81.9
2	47.7	3.3	44.4	93.1
3	10.7	1.2	9.5	88.7
4	23.7	15.3	8.5	35.6
5	34.3	8	26.3	76.7
6	18.3	11.3	6.9	37.9
7	4.4	1.6	2.8	64.6
8	12.7	0.7	12.1	94.7
9	8.3	0.6	7.7	92.5
10	23	6.8	16.2	70.5
11	16	4.2	11.8	74
12	21.1	7.9	13.2	62.4
13	10.8	2.8	8.1	74.4
14	67.6	41.4	26.3	38.8

## Discussion

In the present study, it was observed that the preoperative intravitreal administration of rTPA one day before surgery and then performing a vitrectomy with a subretinal injection of rTPA and an air tamponade reduced the submacular hemorrhage area significantly (p=0.02) by around 70.4%. There was a significant improvement in visual acuity (p=0.01) by 31.7%, and a significant reduction of a central macular thickness (p=0.03) by 38.1%. SMH is a common symptom associated with neovascular age-related macular degeneration.

SMH causes the degeneration of the photoreceptor cells in the retina. This SMH-induced degeneration is due to iron toxicity, retinal contraction caused by the shrinkage of the fibrin meshwork, and reduced nutrient supply to the retina, which leads to scar formation in the macula [1]. Iron derived from the clot or accumulated blood directly causes toxicity in the photoreceptors carried out by the free radicals generated by the iron through the Fenton reaction [10]. SMH acts as a barrier in the trafficking of nutrients between the retina and RPE, resulting in reduced nutrient supply to the retina [11-12]. The accumulated fibrin network within the clot contracts and causes shrinkage and damage to the photoreceptors [13]. The extent of SMH induced damage depends on the duration of the formation of the SMH. The more the duration of SMH, the higher the damage to the retina [14]. Studies in animal models have reported that the retinal damage induced by SMH is irreversible and occurs within 24 hours, which subsequently leads to complete loss of photoreceptors [15].

Since the degenerative effects of the SMH start as early as 24 hours, timing is very important while treating SMH. Any delay beyond 24 hours may lead to irreversible damage to the sensory cells of the retina. Although the mechanical removal of SMH has several negative effects on the retina, such as retinal detachment (RD) and proliferative vitreoretinopathy, the use of rTPA has improved the situation drastically.

An array of treatments exists to manage SMH formed secondary to neovascular AMD, which includes the administration of only rTPA or anti-VEGF drugs, or performing vitrectomy with gas tamponade, or a combination of them.

TPA is a thrombolytic serine protease that activates plasminogen to plasmin that eventually dissolves the clot by cleaving fibrin in the clot [16]. So, TPA administered either through intravitreal or subretinal routes in combination with surgery or gas facilitates the removal of the SMH (clot) and reduces the photoreceptor damage, thereby decreasing the iron toxicity and removing the diffusion barrier induced by the clot. Thus, the administration of TPA decreases the loss of visual acuity by decreasing fibrin-mediated photoreceptor damage [16].

Intravitreal TPA can cross the retina to exhibit its thrombolytic effects in dissolving the SMH [17]. It has been reported that TPA, when injected intravitreally, causes the disappearance of the clot in the subretinal region within 24 hours [18].

It has been shown that an intravitreous TPA (50 mg) injection, 12 to 36 hours prior to surgical removal of the blood, resulted in complete liquefaction of SMH [19]. TPA has been reported to be safely used to treat SMH at a dose of 50 mg or less [20]. Higher doses (100 mg) of TPA may lead to retinal toxicity, exudative retinal detachment, RPE hyperpigmentation, distorted B-wave on electroretinography, vitreous hemorrhage, transiently raised IOP, lens trauma, and endophthalmitis [21].

A very short half-life (five minutes) of TPA reduces the chances of causing toxicity and interaction with other drugs [22]. Intravitreous TPA combined with gas has been successful in removing the SMH. In this combined technique, TPA dissolves the clot and the gas bubble mechanically displaces the liquefied blood [23].

Vitrectomy combined with TPA and blood removal is another common way of managing SMH. Here also TPA is used as an adjunct to liquefy the clot and facilitate the removal of the blood by vitrectomy [24]. In this process, the use of TPA avoids the necessity of clot/thrombus removal with forceps by creating large retinotomies. This procedure reduces the risk of iatrogenic RD and PVR and causes less damage to the photoreceptors during clot removal [24]. When further combined with gas, vitrectomy and subretinal TPA administration avoids the mechanical removal of the clot [9].

Studies have also shown that the vision is related to the amount, location, and extension of the hemorrhage [25].

De Silva et al. mentioned that early treatment of submacular hemorrhage using intravitreal TPA, C3F8, and anti-VEGF was effective in significantly improving visual acuity in this series of patients who presented soon after symptom onset. Treatment was well tolerated in this group of elderly and potentially frail patients [26]. Multiple subsequent reports demonstrated visual gains [27-28]. Alternatively, TPA may be administered via vitrectomy and subretinal injection with or without expansile gas [29]. A recent review concluding that the treatment of submacular hemorrhage with vitrectomy, subretinal TPA, intravitreal gas, and anti-VEGF therapy resulted in the greatest visual improvement [30].

In view of the short timescale within which photoreceptor damage occurs and the significant effect of the use of less invasive intravitreal injection of rTPA in dissolving the blood in SMH, and the complications associated with surgical removal of subretinal blood clots, we wanted, in this case series, to evaluate the efficacy of using a combined treatment of 24 hours preoperative intravitreal injection of rTPA with a 23G ppV and subretinal rTPA injection with mechanical displacement of the accumulated submacular blood, with an air tamponade as a prompt intervention of patients presenting with acute SMH. Although it is a more invasive intervention as compared to the other existing less invasive treatments of acute SMH secondary to neovascular AMD, it appeared to be effective in improving the visual acuity and reducing the SMH area significantly.

In this case series, we also used a lower dose of rTPA (intravitreal and subretinal) and observed that at such low doses, rTPA was also able to dissolve the blood clot without any side effects or complications. To the best of our knowledge, this is the first study of its kind where the preoperative intravitreal administration of rTPA has been evaluated in combination with vitrectomy and the subretinal administration of rTPA with the mechanical displacement of the SMH using an air tamponade.

## Conclusions

Combined treatment of 24 hours preoperative administration of intravitreal rTPA followed by vitrectomy and the administration of subretinal rTPA with air tamponade was found to have a significant effect on managing acute SMH secondary to neovascular AMD. This study, owing to its small sample size and limited follow-up, has certain limitations to generalize these findings. Further studies/trials with larger sample sizes and control comparative group/s are recommended to further validate these findings and may yield more useful information.
